# On the relevance of task instructions for the influence of action on perception

**DOI:** 10.3758/s13414-021-02309-x

**Published:** 2021-04-30

**Authors:** Wladimir Kirsch

**Affiliations:** 1 Department of Psychology, University of Würzburg, Würzburg, Germany; 2 Institut für Psychologie III der Universität Würzburg, Röntgenring 11, D-97070 Würzburg, Germany

**Keywords:** Perception and action, Multisensory processing

## Abstract

The present study explored how task instructions mediate the impact of action on perception. Participants saw a target object while performing finger movements. Then either the size of the target or the size of the adopted finger postures was judged. The target judgment was attracted by the adopted finger posture indicating sensory integration of body-related and visual signals. The magnitude of integration, however, depended on how the task was initially described. It was substantially larger when the experimental instructions indicated that finger movements and the target object relate to the same event than when they suggested that they are unrelated. This outcome highlights the role of causal inference processes in the emergence of action specific influences in perception.

**Supplementary Information:**

The online version contains supplementary material available at 10.3758/s13414-021-02309-x.

## Introduction

Anatomical and movement-related features of the human body affect the perception of environmental objects and events in space (e.g., Witt, 2011) and time (e.g., Haggard et al., 2002). The traditional accounts of these influences emphasize motor processes, such as those related to action ability or to the sense of agency (e.g., Haggard, 2017; Witt, 2011), or consider body as a central reference for sensory input (e.g., Harris et al., 2015; Proffitt & Linkenauger, 2013; see also Scheerer, 1984, for a historical review). However, to date it is unclear whether such influences are somehow unique, or instead reflect well-known principles of sensory integration of multimodal signals that body-environment interactions necessarily include as well (e.g., Debats et al., 2017a; Kawabe et al., 2013; Kirsch et al., 2017, 2019; Wolpe et al., 2013; Yamamoto, 2020).

Consider, for example, the phenomenon of spatial attraction between actions and their effects that we originally termed “spatial binding” in analogy to a related phenomenon in the temporal domain known as “temporal binding” (Kirsch et al., 2016). In cursor-control tasks, the perception of a visual cursor is attracted by the position and direction of the hand controlling it when a visual-proprioceptive conflict is introduced. Simultaneously, the perception of the hand is attracted by the position and direction of the cursor (e.g., Rand et al., 2013; Rand & Heuer, 2013, 2016). The latter bias is usually larger than the former, but the relative magnitude of both biases depends on the relative reliability of visual and body-related information (Debats et al., 2017a; see also, e.g., Yamamoto, 2020, for related results in the temporal domain). These observations are well explained by a basic multisensory principle suggesting that a multimodal percept is a weighted average of unimodal signals and that the weight of each signal depends on its reliability (e.g., Ernst & Bülthoff, 2004).

These findings cover situations in which features of external objects are a direct consequence of actions and suggest that body-related and environmental action effects are combined in perception. However, in many situations we act in a way that would suit possible interactions with environmental objects without actually changing these objects, while action might still affect perception of those objects according to previous research (e.g., Witt, 2011). For example, imagine you tried to communicate the size of a somewhat distant box to someone else by holding your hand at a distance that mimics that box size. Would features of the box be integrated with your hand action, although the box is not changed in any respect here? The results of our prior studies suggested that action and object features are in fact integrated under such conditions (see also Takahashi et al., 2009; Takahashi & Watt, 2014, 2017). In a virtual grasping task that we used in a previous study, participants enclosed visual objects by manually controlled visual cursors. The current hand opening attracted the judgments of an object’s size and vice versa, the size of the visual object attracted the judgments of the hand opening (Kirsch et al., 2017). Moreover, in accordance with the reliability weighting principle, the former bias increased and the latter decreased when the reliability of the visual information decreased (Kirsch & Kunde, 2019a, 2019b).

In the present study we aimed to go one step further and explored another basic principle of multisensory integration, the so-called “unity assumption.” This principle holds that signals are integrated to the extent that they relate to the same object or event, i.e., to the extent that they are redundant (Chen & Spence, 2017; Welch & Warren, 1980). It implies that the perceptual system has to determine whether signals belong together before integration. This “correspondence problem” is solved in a top-down manner based on the knowledge about the co-occurrence or correlation of the signals. The more the observer believes that the signals relate to the same event, the stronger multimodal integration is (see also, e.g., Deroy et al., 2016; Ernst, 2006; Roach et al., 2006; Shams & Beierholm, 2010).

**Fig. 1:**
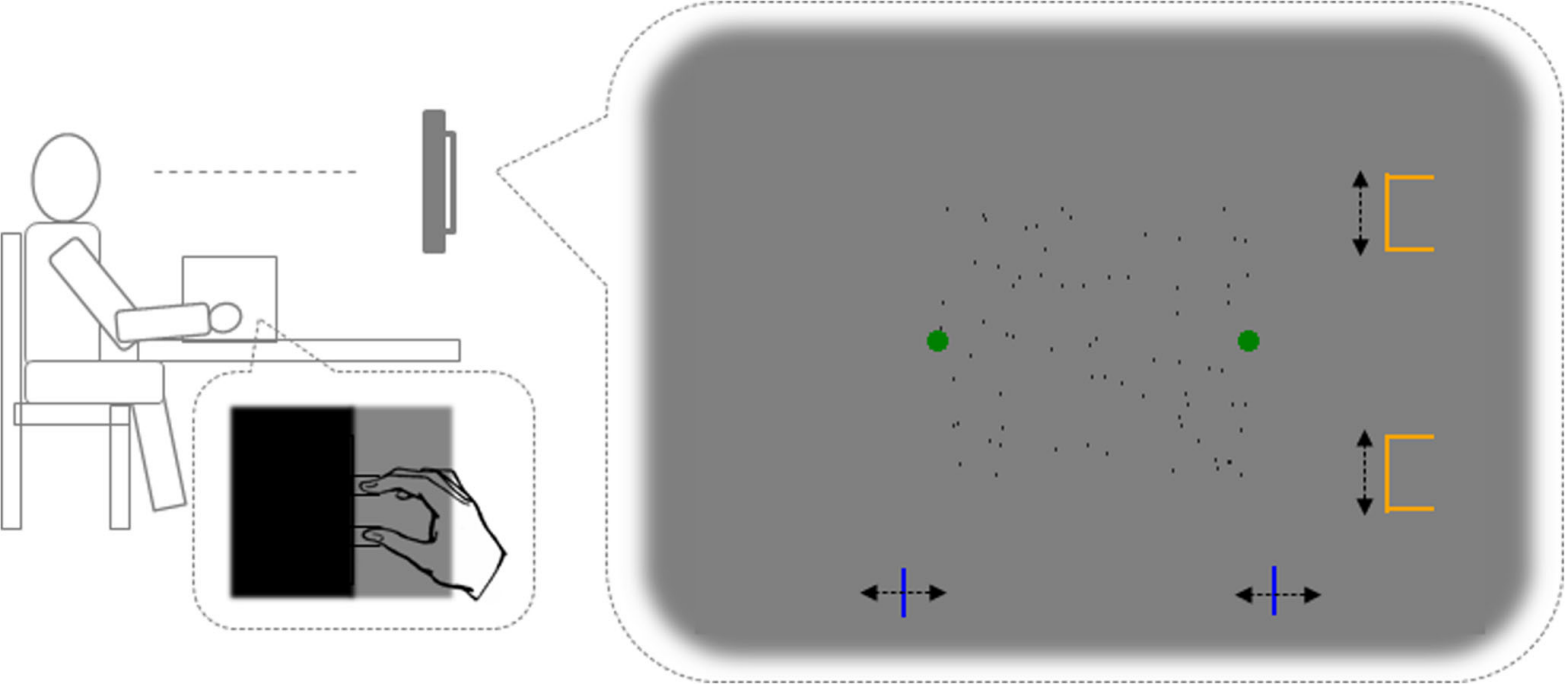
Experimental setup (**left side**) and visual objects presented on the screen (**right side**). Stimuli are not drawn to scale. *Note.* Neither the blue nor the orange objects were controlled by the finger opening. The blue lines served to make object-width estimates, while the orange objects served to make finger-opening estimates. Both were controlled by keypresses of the left hand

Predictions derived from this principle have already been approved in the context of actions in the cursor-control task (Debats et al., 2017b; Debats & Heuer, 2018; Debats & Heuer, 2020a, 2020b). In the present study, we tested whether it holds for interactions with distant objects, which, however, are not affected by the action. We used a version of the virtual grasping task and experimentally varied the task instructions given to the participants (cf. Fig. 1). All participants saw a rectangular object with two dots at its side and moved their fingers to certain positions until an acoustic signal was presented and the dots disappeared. For one group of participants, this task was described as a virtual grasping. That is, participants were basically asked to virtually grasp the rectangular object by the (invisible) cursors of their finger movements. Another group of the participants received a sound production instruction. This group was asked to produce a sound with finger movements. The rationale was as follows. The instruction suggests that finger movements and the visual target object relate to the same event in the virtual grasping group, but to different events in the sound production group. Accordingly, the magnitude of integration (i.e., integration strength) should be larger for the former than for the latter instruction.

The adopted finger distance varied around the width of the rectangle. This constituted a type of visual-proprioceptive conflict. Participants judged either the adopted finger distance or the width of the rectangle. The overall magnitude of visual-proprioceptive attraction (i.e., the sum of the bias towards the fingers in the width judgments and of the bias towards the rectangle’s width in the finger judgments) served as a measure of integration strength (see, e.g., Debats & Heuer, 2020a). Note that the strength of integration can range from a complete fusion of the signals into a single percept to a complete independence (e.g., Ernst, 2006). For the present task situation only partial integration is expected that is in-between these two extremes (e.g., Debats & Heuer, 2020b; Kirsch et al., 2017). Also note that there was a certain level of correlation between finger movements and the visual target object in both instruction conditions as larger target objects went along with larger finger distances and due to visual movement effects (i.e., disappearance of dots after a finger distance was adopted). Thus, a certain level of integration was expected for both instruction conditions of the present study.

The idea that cognition in general and task instructions in particular influence the interplay between perception and action is not new and has been already discussed within a related theoretical domain (Ansorge, 2002; Dreisbach & Haider, 2008; Haazebroek, van Dantzig, & Hommel, 2013; Hommel, 1993; Veto et al., 2018). For example, in an auditory Simon task, Hommel (1993) asked participants to press left and right keys in response to the pitch of a tone presented on the left or right side. The key press caused a visual stimulus to appear on the opposite side of the key. A standard Simon effect (better performance when the tone and key location corresponded) was observed when participants were instructed in terms of key locations. This effect was, however, reversed when the instruction stressed the locations of the visual stimulus. To explain this and similar results, it has been proposed that task-relevant features of stimuli and responses are attended (or activated) more strongly than task-irrelevant features before perceptual and action features are bound together into a common cognitive representation known as an “event file” (e.g., Frings et al., 2020; Hommel, 2019; Memelink & Hommel, 2013; Mocke et al., 2020). If a multimodal percept is construed as an event file (Spence & Frings, 2020) and body-related and visual object characteristics as distinct features within this file, the mentioned proposal would predict the same outcome for the present study as the multisensory approach sketched above. That is, less integration between visual and body-related signals can be expected for the sound production condition than for the virtual grasping condition because visual object features are less task relevant in the former than in the latter condition.^1^ Thus, the present setup enabled us to test an additional hypothesis inferred from a related line of research that relies on different methods (especially reaction times) and a different theoretical rationale. I return to this issue in the *Discussion* section.

## Methods

### Participants

Forty-eight right-handed participants with normal or corrected-to-normal vision were recruited. The data collection of one participant had to be aborted for technical reasons. This participant was replaced by another one prior to data screening and analyses. The sample included 41 females and seven males (*M*_*age*_ = 24 years, *SD* = 4). All participants gave their written informed consent for the procedures and received monetary compensation (€8) for their participation. The sample size was determined a priori based on a pilot experiment^2^ and ensured a power of 1−*β* = 0.80 (*α*=.05) for effect sizes of *d* = 0.73 (cf. also *d*_*z*_ of about 0.7 for a related effect in Debats & Heuer, 2020a, and *d* of .75 and of 1.23 in Debats & Heuer, 2018). The study was performed in accordance with the Declaration of Helsinki (1964) and was approved by the local ethics committee (GZ 2019-04).

### Apparatus

The experimental room was dimly lit. Participants were at a distance of approximately 68 cm from the screen (Fujitsu Siemens P19-1; 1,280 × 1,024 pixels; 1 pixel = 0.294 mm; 60 Hz) centered slightly below the eye level. Their head was supported by a chin rest. Headphones were used to present acoustic signals. A finger movement device (see Fig. S1 in the Online Supplementary Materials for more details) was positioned on a table in front of the participants. They placed their index finger and the thumb of the right hand on two moveable U-shaped metal plates, which were mirror-symmetrically interlocked (see Fig. 1). We bound the index and the middle fingers together to make the finger movements more comfortable and to prevent exploratory finger movements.

### Stimuli and procedure

The background of the screen was gray. Each trial started with a pair of dark-gray arrows (5 mm × 0.3 mm) presented in the middle of the screen. The arrows were placed one upon the other and the arrowheads were oriented to each other. This stimulus prompted the participants to move their fingers closer together (until a contact of the plates) to initiate the next trial. The following procedure contained two successive parts – finger movements and perceptual judgments.

- Finger movements: A target object appeared in the middle of the display in the majority of trials (regular trials). This object was composed of 69 dark gray dots (0.3 mm × 0.6 mm in size) randomly distributed along a rectangular shape. Simultaneously, two green circles (about 2.5 mm in diameter) appeared at the left and right edges of the rectangle (see Fig. 1). In response to this stimulus, participants had to move their fingers apart until the circles disappeared and a clicking sound was presented. This happened when a certain finger distance was reached (see *Design*). In particular, the circles disappeared/sound was presented when the current finger distance deviated by less than 5/2 mm from the required finger distances. Participants had to maintain this finger posture for about 1.5 s and to perform corrective movements when the sound disappeared (i.e., when the deviation from the required finger distance exceeded 2 mm). In some trials (control trials), no rectangle was presented. Instead, a German word for “search” appeared. The task was basically the same as in the regular trials, i.e., to move the fingers until a certain finger distance, indicated by a clicking sound, was reached. These control trials were included to assess a general bias toward mean finger posture unrelated to the virtual interaction with an object (i.e., central tendency effect).
- Perceptual judgments: After a given finger posture was maintained for 1.5 s, the rectangle (or the word “search”) disappeared, and the participants had to estimate either the width of the just-seen rectangle or the current hand opening (i.e., the distance between the thumb and the index (and middle) finger. For this purpose, either a pair of blue lines (7 × 0.6 mm; about 7 cm below the screen center) or a pair of U-shaped line objects (7 × 11 × 7 mm; about 9 cm to the right of the screen center; see Fig. 1) were presented, respectively. In addition, the German word for “width” (in blue)/“finger” (in orange) appeared in the upper part of the screen together with the blue lines/orange objects (not shown in Fig. 1). Participants pressed the left/right button of a computer mouse with their left hand to increase/decrease the distance between these stimuli and the middle mouse button (mouse wheel) to confirm the judgment. The initial distance between the lines/U-shaped objects corresponded either to 50% (half of trials) or to 150% (another half of trials; random order) of the width of the rectangle/of the actual finger distance (measured between the inner plates of the movement device).

Error feedback appeared and the trial was immediately repeated, when (a) participants changed the opening of the right hand during the judgments, or (b) when the left or right mouse buttons were pressed during the finger movement part, or (c) when the middle mouse button was pressed before the initial position of the lines or of the U-shaped line objects was changed.

### Design

There were four blocks of trials in the main experiment. Each block included 48 trials. In each block, each participant was exposed twice to each combination of two different rectangles (37 × 43 mm and 43 × 37 mm), three different types of judgment (width of rectangle, finger distance in the regular trials, finger distance in the control trials), and four different finger distances corresponding to 0.4, 0.8, 1.2, and 1.6 of the width of each rectangle. The order of these conditions was random. The main experiment was preceded by six practice trials that were not included in the analyses.

The critical experimental manipulation was related to the description of the finger movements provided to the participants before the experiment. For one-half of the participants, the task was described as a grasping task, suggesting that finger movements relate to the visual target object. In particular, the participants were asked to enclose the rectangle with (invisible) cursors controlled by their finger movements. The acoustic signal served as a feedback for the correct finger distance (i.e., for the correct grasping of the object). The written instructions contained the following information (bold type and color as presented): “Initially, you have to **virtually grasp** an object consisting of dots. Your finger movements refer to the **horizontal** dimension of the object and the **green** dots indicate the grabbed edges of the object. Reaching the right finger position is indicated by a **clicking sound**. Maintain this finger posture.” For another half of the participants, the instruction did not contain a link between finger movements and the visual target object. That is, the finger movements and the rectangle were described as unrelated by omitting any links to grasping. Thus, the task was basically to move the fingers until the presentation of the acoustic signal (i.e., to produce this signal by finger movements). The corresponding part of the instructions reads: “You will initially see an object consisting of dots and including two **green** dots. You have to produce **clicking sound** by the movements of your right hand that causes the **green** dots to vanish. Maintain the finger posture at which the sound is continuously presented.” Participants were randomly assigned to one of these instruction conditions.

### Data analyses

We initially computed a difference score between the actual and the estimated value of spatial extent for each trial (judgment error). Positive/negative errors reflect, by definition, overestimation/underestimation. These values were then averaged over repetitions for each participant and each experimental condition. The mean judgment errors entered subsequent linear regression analyses as a dependent variable (e.g., Debats et al., 2017b). These analyses were performed for each rectangle, each judgment type, and each participant, and the introduced visual-proprioceptive discrepancy was used as a predictor variable. The slope parameters of the regression equations (unstandardized regression coefficients) provided information about the impact of the rectangle on finger perception in case of finger judgments and, vice versa, of the finger posture on rectangle perception in case of rectangle judgments. For the finger judgments, slope values observed in the control trials were subtracted from the slope values observed in the regular trials in order to correct for any effects unrelated to virtual interactions with the rectangle before statistical analyses. The overall magnitude of inter-sensory coupling, that is the integration strength, is then given by a difference^3^ between slope values observed in judgments of the rectangle and those observed in the judgments of the fingers (“bias to fingers” minus “bias to object”). A value of 1 indicates an identical multimodal percept for finger and rectangle judgments, i.e., sensory fusion. A value of 0 indicates sensory independence and no mutual attraction between visual and body-related signals. Any other values in between suggest partial integration.

The critical test of the proposed hypothesis was performed using an unpaired t-test that compared the mean integration strength of the two groups exposed to different task instructions. In addition, analyses of variance (ANOVAs) were run on slope parameters for finger and rectangle judgments separately to validate the main results (with instruction, type of judgment (including two levels of rectangle judgment and corrected finger judgments), and rectangle size as within-subject factors and instruction as a between-subject factor). We also ran one-sample t-tests against zero on the slope parameters to test whether the rectangle and finger judgments were significantly affected by the visual-proprioceptive discrepancy in each instruction condition.

The raw data have been made publicly available (https://osf.io/cwmg8/).

## Results

**Fig. 2:**
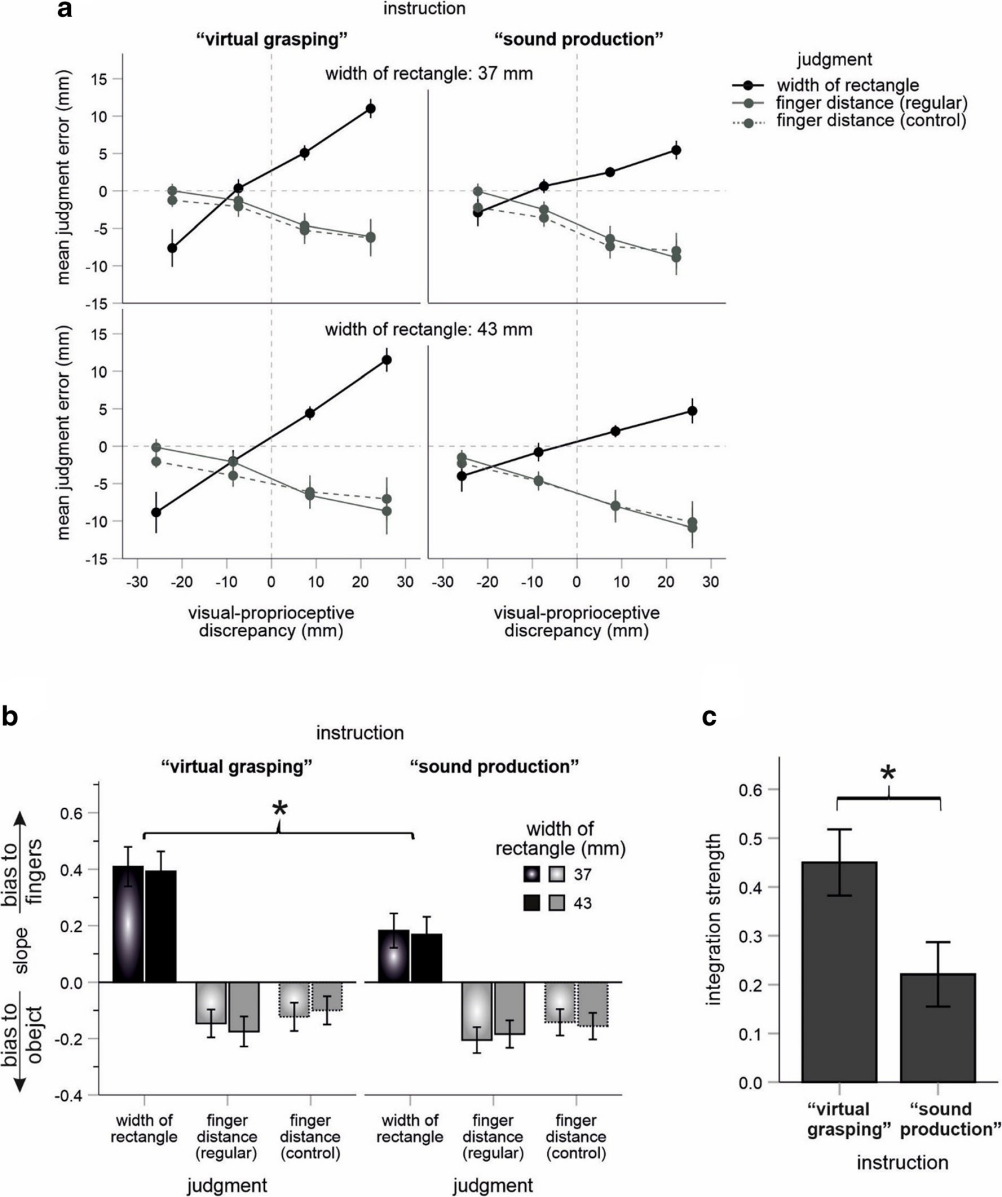
Main results. (**A**) Mean judgment errors. Negative/positive visual-proprioceptive discrepancies indicate finger distances that are smaller/larger than the width of the rectangle. (**B**) Mean slope coefficients from the regression of judgment errors on the four visual-proprioceptive conflict conditions for each instruction, each rectangle, and each judgment condition. (**C**) Integration strength for the two task-instruction conditions. Error bars are standard errors. Asterisks denote statistical significance (p < .05)

Figure 2A illustrates the mean judgment error in all experimental conditions. The error increased with an increase of the introduced visual-proprioceptive conflict for rectangle judgments and decreased for the finger judgments. This pattern indicated perceptual attraction of the rectangle judgments by the adopted finger distance and of the finger judgments by the rectangle (see also, e.g., Kirsch & Kunde, 2019b). However, the latter bias (“bias to object”) appeared to be rather small as the slope of the control condition is very similar to that observed in the regular finger-judgment trials. In contrast, the impact of the finger distance on rectangle judgments (“bias to fingers”) was strongly pronounced, and, more importantly, was larger when the task was instructed in terms of virtual grasping than when sound production was stressed. These observations were supported by statistical analyses.

The integration strength was significantly larger for the “virtual grasping” instruction than for the “sound production” instruction, as predicted, *t*(46) = 2.42, *p* = .019, *d* = .70 (see Fig. 2C). This effect was basically due to a larger impact of finger distance on rectangle judgments: an ANOVA on slope values of rectangle judgments revealed a significant main effect of instruction, *F*(1, 46) = 5.93, *p* = .019, *η*_*p*_^*2*^ = .114 (other p > .284; see black bars in Fig.2B). The slopes were significantly different from zero in all rectangle judgment conditions (all *p* ≤ .014). Another ANOVA on (corrected) slope values of finger judgments revealed a marginally significant interaction between instruction and rectangle size, *F*(1, 46) = 3.78, *p* = .058, *η*_*p*_^*2*^ = .076 (other *p* > .705; see gray bars in Fig. 2B). This small trend arose because the bias tended to be larger for the wide than for the narrow rectangle in the virtual-grasping group and the other way around in the sound-production group (pairwise comparisons did not reveal any significant results, all *p* > .123). Also, the corrected slopes were significantly different from zero for the wide (*t*(23) = 3.62, *p* = .001) but not for the narrow (*t*(23) = .82, *p* = .421) rectangle in the virtual-grasping group, and the other way around in the sound-production group (*t*(23) = 3.17, *p* = .004; *t*(23) = 1.31, *p* = .202).

## Discussion

The present study tested whether perceptual changes observed with spatiotemporal contiguous actions obey the unity assumption. This basic principle of multimodal integration holds that the strength of intersensory coupling depends on the extent observers believe that signals relate to the same event. The results were in accordance with this assumption. Integration strength was larger when the experimental instruction emphasized that action and visual object features relate to the same event than when it suggested that action and visual object are unrelated. This outcome further strengthens the multisensory view on actions’ effect on perception suggesting that the perceptual biases observed in the context of actions reflect resolutions of experimentally introduced cross-modal conflicts (Kirsch et al., 2017; Kirsch & Kunde, 2019a, 2019b). According to this approach, action provides body-related afferent signals that inform about external events (and about own body) like other sensory modalities. There is, thus, no need to postulate a special function for the motor system or the body here beyond producing perceptions (see also *Introduction*).

This claim is reminiscent of the so-called common coding framework suggesting that action and perception share common cognitive representations (e.g., Hommel et al., 2001; 2019; Prinz, 1997; see also Frings et al., 2020). The multisensory perspective is not in conflict with this framework, but extends it by a few approved principles according to which these representations emerge and thus enables more detailed and quantifiable assumptions about underlying cognitive mechanisms. For example, it has been suggested that feature dimensions can be differently weighted (i.e., attended or activated) and can thus have a varying impact on event representations depending on their relevance for a given task (Memelink & Hommel, 2013; Mocke et al., 2020; see also *Introduction*). The unity assumption discussed in the classical multisensory research can help here to specify in more detail when, how, and to what extent this should take place. In particular, the more reasons a given task context conveys to believe that signals (or features) belong together, the stronger the proposed “intentional weighting” should be. Moreover, the reliability weighting principle enables better characterization of the nature of the proposed event representation (i.e., event file) insofar as it predicts the relative impacts of different information sources on the overall representation based on information quality (i.e., reliability). This could relate to the extraction of a single feature such as “object size” based on body-related and visual signals as in the present study. The same principle could in theory also operate on the level of different features such as “size” and “weight” as long as they are experienced as interrelated. Both causal processes and reliability weighting have been formalized within a Bayesian framework (e.g., Ernst, 2006; Shams & Beierholm, 2010). This allows testing of not only qualitative but also quantitative predictions. To explore these potential links between related but widely independent approaches might be interesting for future research and could provide deeper insights into the interplay between perception and action.

There has been intense debate about whether action truly alters perception. One prominent argument against this claim was based on the impact of experimental instructions (e.g., Firestone, 2013; Firestone & Scholl, 2016). It has been observed that some action effects disappeared with changes in the description of the task. For example, an effect of wearing a backpack on the perception of hills (Bhalla & Proffitt, 1999) vanished when the instructions provided a fictitious explanation for the backpack’s presence (Durgin et al., 2009; for related findings, see also Durgin et al., 2012; Firestone & Scholl, 2014; Shaffer et al., 2013; Wesp & Gasper, 2012; Williams et al., 2012). The authors argued that this effect should thus reflect changes in judgment behavior rather than in perception. Even though plausible at first glance, such a conclusion could be premature given the multisensory approach and the present results. Instructing the participants in a way that a body-related variable (i.e., backpack) has nothing to do with the object being judged can bias participants’ inference processes accordingly. As a result, an effect that occurs under more natural conditions (i.e., without explicit instructions) might disappear due to the weakening of the coupling strength rather than due to the weakening of a judgment or response bias. The results of one of our earlier studies, in which we observed a backpack-like effect in reaching movements and where an impact of a simple judgment bias appears unlikely, point in this direction (Kirsch & Kunde, 2013; cf. also the “sound-production” condition of the present study).

Appling the principles of multisensory integration in the context of actions does not only explain the emergence of body-related influences in visual judgments, but also of visual influences in body-related judgments. In the original version of our virtual grasping task, for example, in which finger movements are accompanied by movements of visual cursors, the latter bias is usually larger than the former, indicating a larger impact of visual than of body-related information in the multimodal percepts (e.g., Kirsch & Kunde, 2019b; see also, e.g., Debats & Heuer, 2020b, for related observations in a cursor-control task). In the present study, however, the impact of visual object information was rather weak and partly non-significant. We assume that using stationary dots (green dots in Fig. 1) instead of movable cursors substantially weakened the impact of visual information. Accordingly, the overall integration strength was determined mainly by body-related information.

To sum up, the present study suggests that the impact of action on perception depends on task instructions that influence participants’ knowledge about event occurrence and thus their willingness to bind these events in perception in order to increase its precision.

## Supplementary Information

ESM 1 (DOCX 567 kb)

## Footnotes

1 In other words, the visual feature would be more strongly activated and thus enter the crucial event file (i.e., the representation of the event “object”) with a higher probability in the virtual grasping than in the sound-production condition. Note, however, that one could also argue within this framework that body-related and visual object signals constitute one single feature such as the “object’s width.” In this case, no straightforward predictions are possible for the present task situation. Also note that “task-relevance” here is defined in terms of the relevance for the perception of the target object (i.e., for the event file) and all other aspects of the task are identical for both instruction conditions.

2 This experiment was very similar to the present one. The main difference was a smaller sample size (N=24) and one additional manipulation of visual reliability that was omitted in the present experiment. Analyses revealed an effect size of about 0.7 for the critical difference in the impact of finger distance on the visual judgments between the instruction conditions (cf. Fig.2C). We reasoned that doubling the sample size of the pilot study should be sufficient to ensure that this effect exists.

3 Not a sum because of the inverse sign applied to the “bias to object” for illustrative purposes under the present conditions (see Fig. 2).
