# Supplementary material for: On the relevance of task instructions for the influence of action on perception
Source: Atten Percept Psychophys. 2021 Apr 30;83(6):2625–33. doi: 10.3758/s13414-021-02309-x (PMC8302516; doi:10.3758/s13414-021-02309-x)
Supplement: Supplementary file 1 — (DOCX 567 kb) [file 13414_2021_2309_MOESM1_ESM.docx]

Supplementary materials


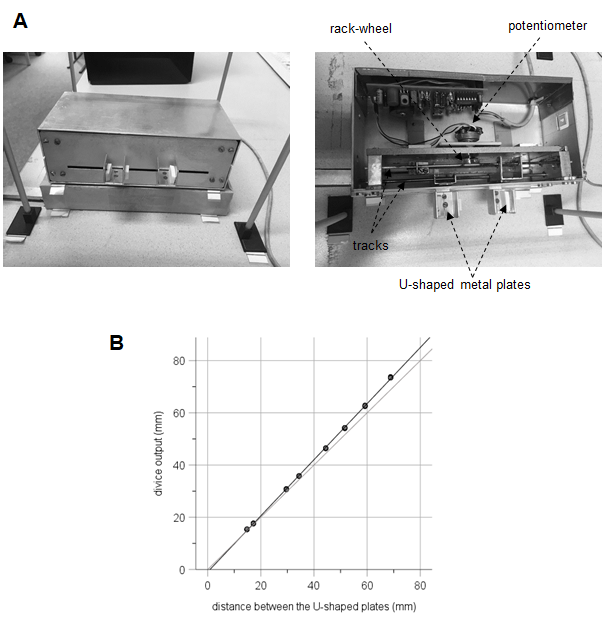


Figure S1. Finger movement device. (A) Participant manually operated two U-shaped metal plates, which moved along two separate tracks. Each metal plate was connected to a strap than put a plastic rack-wheel in motion. The rack-wheel was attached to a potentiometer by means of which the motion of the rack-wheel was transformed into the position signals of the metal plates. (B) A series of measurements indicating the accuracy of the device. The metal plates were adjusted to eight distances used in the present study (five times each) by means of a digital measuring slide (X-axis) and the device output was measured (Y-axis). The device had a high spatial resolution (below 0.5 mm), but a small constant error (slight increase in overestimation with an increase in distance) that can be assumed to have no substantial impact on the results.
